# Commencement of the New York College of Dental Surgery

**Published:** 1853-04

**Authors:** 


*Commencement of the New York College of Dental Surgery.*—We copy from the Dental News Letter the following account of the commencement of the above Institution, held on Tuesday, March 1st, 1853:

The valedictory address was delivered by Prof. Stevens, upon the "Condition of Professional Science." It was replete with sound views and useful suggestions, and was listened to with attention and pleasure by the audience.

The graduating class consisted of young gentlemen who had completed the course of study required by the charter, and who were recommended by the examining committee as suitable persons to receive the degrees of "Doctor of Dental Surgery."

Diplomas were conferred by Dr. Westcott, the Dean of the Faculty, upon B. F. Wright, L. G. Bartlett, W. W. Alport, I. D. Kilbourne, B. C. Lefler, W. Dalrymple.

Dr. Hervey, of Buffalo; Dr. Dunning, of New York; and Dr. Nelson, of Albany, the examining committee, were present.

We are gratified to learn of the success of the New York Dental College, in the second year of its existence. It is the only institution of the kind in the State, and must powerfully contribute to elevate the standard of excellence in the dental profession, and diffuse a more thorough acquaintance with dental science among the profession.

Heretofore, in this State, dental surgery has only been taught in private offices. The consequence has been, that the advantages for obtaining a thorough and complete education as a dental surgeon, have been restricted.

The New York Dental College has a very able corps of Professors, is well supplied with anatomical and dental preparations and collections for illustration, and every facility is furnished to the dental student for obtaining a thorough knowledge of the theory and practice of dental surgery.

Prof. Parmly, we learn, is soon to return from Paris, when he will enter upon the active duties of his professorship in this school. Our citizens may well feel interested in the success of the New York College of Dental Surgery.

